# Altered Glutamatergic Metabolism Associated with Punctate White Matter Lesions in Preterm Infants

**DOI:** 10.1371/journal.pone.0056880

**Published:** 2013-02-26

**Authors:** Jessica L. Wisnowski, Stefan Blüml, Lisa Paquette, Elizabeth Zelinski, Marvin D. Nelson, Michael J. Painter, Hanna Damasio, Floyd Gilles, Ashok Panigrahy

**Affiliations:** 1 Department of Radiology, Children's Hospital Los Angeles, Los Angeles, California, United States of America; 2 Brain and Creativity Institute, University of Southern California, Los Angeles, California, United States of America; 3 Department of Pediatrics, Division of Neonatology, Children's Hospital Los Angeles, Los Angeles, California, United States of America; 4 Davis School of Gerontology, University of Southern California, Los Angeles, California, United States of America; 5 Department of Pediatrics, Division of Neurology, Children's Hospital of Pittsburgh of UPMC, Pittsburgh, Pennsylvania, United States of America; 6 Department of Pathology and Laboratory Medicine, Neuropathology Section, Children's Hospital Los Angeles, Los Angeles, California, United States of America; 7 Department of Radiology, Children's Hospital of Pittsburgh of UPMC, Pittsburgh, Pennsylvania, United States of America; Hôpital Robert Debré, France

## Abstract

Preterm infants (∼10% of all births) are at high-risk for long-term neurodevelopmental disabilities, most often resulting from white matter injury sustained during the neonatal period. Glutamate excitotoxicity is hypothesized to be a key mechanism in the pathogenesis of white matter injury; however, there has been no *in vivo* demonstration of glutamate excitotoxicity in preterm infants. Using magnetic resonance spectroscopy (MRS), we tested the hypothesis that glutamate and glutamine, i.e., markers of glutamatergic metabolism, are altered in association with punctate white matter lesions and “diffuse excessive high signal intensity” (DEHSI), the predominant patterns of preterm white matter injury. We reviewed all clinically-indicated MRS studies conducted on preterm infants at a single institution during a six-year period and determined the absolute concentration of glutamate, glutamine, and four other key metabolites in the parietal white matter in 108 of those infants after two investigators independently evaluated the studies for punctate white matter lesions and DEHSI. Punctate white matter lesions were associated with a 29% increase in glutamine concentration (*p* = 0.002). In contrast, there were no differences in glutamatergic metabolism in association with DEHSI. Severe DEHSI, however, was associated with increased lactate concentration (*p* = 0.001), a marker of tissue acidosis. Findings from this study support glutamate excitotoxicity in the pathogenesis of punctate white matter lesions, but not necessarily in DEHSI, and suggest that MRS provides a useful biomarker for determining the pathogenesis of white matter injury in preterm infants during a period when neuroprotective agents may be especially effective.

## Introduction

Preterm birth is a significant challenge in clinical medicine, with nearly 10% of all live births worldwide occurring before 37 weeks gestational age [1]. Each preterm infant is at risk for life-long neurodevelopmental disabilities, including cerebral palsy and cognitive and behavioral impairments, with the highest risk falling among infants born between 23 and 32 gestational weeks [2]. The predominant neuropathological abnormality identified in preterm neonates is white matter injury (WMI), characterized by necrotic foci in the periventricular white matter and surrounding gliosis, historically labeled periventricular leukomalacia (PVL), and/or diffuse white matter gliosis, characterized by reactive astrocytes and activated microglia without necrotic foci and historically labeled perinatal telencephalic leukoencephalopathy [3], [4], [5]. Although published data demonstrating precise radiological-pathological correlations are limited, the punctate T1-hyperintense white matter lesions (pWMLs) visualized on magnetic resonance imaging (MRI) suggest focal necrosis with lipid-laden macrophages, i.e., organizing necrosis in the subacute stage [6], [7], [8], [9], [10]. On the other hand, diffuse excessive high signal intensity (DEHSI) visualized on T2-weighted images, is thought to reflect diffuse white matter gliosis, also a subacute process requiring days to develop [5], [7], [11], [12]. Considerable experimental evidence suggests that glutamate toxicity caused by cerebral ischemia and/or infection/inflammation plays a key role in the pathogenesis of preterm WMI, particularly pWMLs [3], [5], [13], [14], [15], [16], [17], [18], [19]. However, to date, no study has directly assessed glutamate homeostasis in the white matter of premature infants *in vivo*—a serious gap in knowledge because it limits the relevance of extrapolations of the demonstration of glutamate toxicity in experimental and animal paradigms directly to human pathology, as well as translational research towards the establishment of biomarkers and drug treatments.

Under normal physiological circumstances, glutamate, the major excitatory neurotransmitter in the brain, is released from an axon terminal where it interacts with glutamate (NMDA and non-NMDA) receptors on the next neurons in the circuit resulting in calcium influx, membrane depolarization, and neuronal excitation [15]. To prevent further depolarization and maintain extracellular glutamate concentration at sufficiently low levels to prevent excitotoxicity and maintain optimal neurotransmission, glutamate is removed from the synapse by astrocytes expressing high affinity glutamate transporters (Glt1 and GLAST), where it is converted into the non-toxic glutamine [15], [20], [21], [22]. Glutamine, released by astrocytes, in turn, traverses back across the synaptic cleft, is taken up by neurons and converted to glutamate by glutaminase. From there, glutamate may be re-loaded into synaptic vesicles, converted into γ-aminobutyric acid (GABA) or converted into alpha-ketoglutarate and metabolized for energy [15], [20], [21], [22]. Under normal circumstances, glutamine is the most abundant amino acid in the extracellular space, allowing for the recycling of the carbon glutamate skeleton without triggering neuronal depolarization. Indeed, the cycling between glutamate and glutamine, which can adapt to changes in substrate concentration, is an essential component of glutamate homeostasis [20], [21], [22]. In excitotoxicity, the acute extracellular accumulation of glutamate (due to ischemia and/or inflammatory-induced failure of cell membrane pumps, reverse glutamate transport, and other mechanisms) leads to prolonged NMDA and non-NMDA receptor stimulation on adjacent neurons, and especially, pre-myelinating oligodendrocytes, which results in calcium influx and cell damage due to calcium-mediated apoptosis or necrosis, so-called excitotoxicity, that is exacerbated by free radical and cytokine toxicity [15], [20], [21], [22], [23].

Magnetic resonance spectroscopy (MRS) provides the unprecedented opportunity to non-invasively measure glutamate and glutamine concentrations in the brains of living premature infants. While MRS has been utilized to assess injury in the setting of hypoxic-ischemic encephalopathy in human full-term neonates, it has not been utilized to assess the pathogenesis of WMI in preterm infants [24]. Yet, MRS acquired together with conventional MR sequences is clinically feasible in human preterm infants [25], [26], [27], and therefore we utilized it in this study to directly elucidate, for the first time, the potential role of altered glutamatergic metabolism in the pathogenesis of evolving WMI in preterm infants. At present, MRI or MRS is not routinely acquired in the premature infant in the time frame of the acute onset of injury when glutamate levels have been shown to be transiently elevated in experimental models, because the acute injury likely occurs around the time of birth or in periods of marked clinical instability, which are challenging periods for carrying out diagnostic or research neuroimaging. Using *in vivo* MRS, we tested the hypothesis that glutamate and glutamine, i.e., glutamatergic metabolism, are altered subacutely in the parietal white matter of premature infants in the perinatal period in association with white matter injury, and that the pattern of metabolic alteration differs between pWMLs and DEHSI, potentially indicating different factors in their pathogenesis.

## Materials and Methods

### Overview

This study involved a review of all neonatal MRS studies (n = 218) conducted at Children's Hospital Los Angeles (CHLA), a tertiary care hospital, under a single standardized protocol between 2002 and 2008. These infants were screened prospectively as part of on-going longitudinal studies of neurodevelopment in neonates with prematurity. All available cases were included in this study provided: (1) the imaging study had been completed on an infant born before 37 weeks of age; (2) the infant was not older than 60 weeks postconceptional age (PCA: calculated as the interval between the mother's last menstrual period and birth plus postnatal age) at the time of the MRI; (3) an ^1^H MR spectrum was acquired from a single voxel placed in the parietal white matter (standard clinical protocol at this institution on neonatal neuroimaging studies); (4) there was no evidence of cerebral abnormality other than pWMLs or DEHSI (i.e., no large vessel acute or chronic infarction, parenchymal hemorrhage, infection, tumor or cerebral malformation); and (5) there was no clinical or laboratory evidence of liver failure, hyperbilirubinemia (requiring exchange transfusion) or underlying inborn error of metabolism. This study was approved by the CHLA Committee on Clinical Investigations and the University of Pittsburgh Institutional Review Board. Written consents for use of their child's clinically acquired MRI data and for participation in additional neurodevelopmental and neuroimaging studies were obtained from parents on behalf of the prospectively recruited patients by a research coordinator. The ethics committee approved this consent process. Additionally, as this study involved a retrospective review of all clinically-acquired neonatal data for the period between 2002 and 2008, which included neonates who were not enrolled into prospective studies, approval has also been obtained from the CHLA Committee on Clinical Investigations, and the University of Pittsburgh Institutional Review Board for the retrospective use of all clinically-acquired neonatal MRI data obtained at CHLA between 2002 and 2008.

### Case selection

To begin, we searched our database of clinical MRS studies and selected all of the studies acquired from infants born at less than 37 weeks gestational age and scanned no later than 60 postconceptional weeks (n = 218). On first pass, cases were excluded if there was known cerebral pathology other than white matter injury (WMI) recorded in the database. This included cerebral malformations (n = 21), grade III/IV intraventricular hemorrhage (n = 19), stroke (n = 6), hemorrhage (n = 5), tumor (n = 2), congenital infections (n = 5), or other cerebral pathology (n = 12). Cases were included if there was evidence of grade I or II intraventricular hemorrhage, or if there was evidence of cerebellar hemorrhage, which were typically small, punctate hemorrhages. Additional exclusion criteria included clinical or laboratory evidence of liver failure (n = 3), hyperbilirubin requiring exchange transfusion (n = 1), and inborn errors of metabolism (n = 3).

Second, conventional imaging sequences (T1-weighted, T2-weighted, and Diffusion-weighted sequences) for all eligible cases were reviewed by two investigators (JLW, AP). Following the second review, 14 additional cases were excluded due to the presence of other cerebral pathology (9 with evidence of hemorrhage or infarct, 1 with hydrocephalus, 1 with subcortical heterotopic gray matter, 1 with a cerebral malformation, and 2 with calcifications in the posterior limb of the internal capsule, bilaterally). Nine cases did not include data from the standardized parietal white matter location (e.g., due to difficulties during acquisition such as too much infant motion). Finally, 4 additional cases were excluded due to technical difficulties associated with reviewing the conventional MR scans and 6 due to the quality of the MRS spectra (e.g., signal-to-noise (SNR)≤5).

### Determination of pWMLs and DEHSI based on conventional MR images

**Figure 1:**
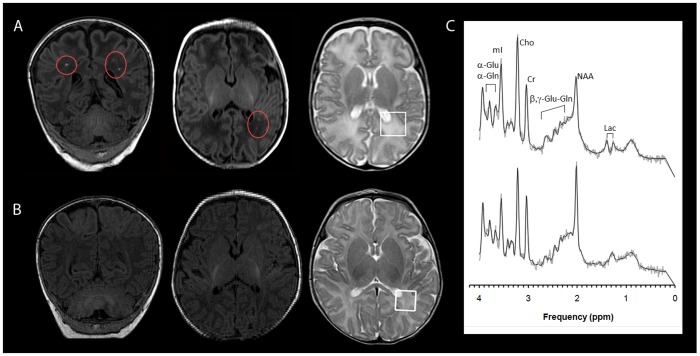
^1^H-MRS spectra were acquired in the parietal white matter. Representative spectra from a neonate (A) with bilateral pWMLs (circled in red) and a neonate (B) without evidence of pWMLs are depicted in panel C (upper spectra was obtained from the 3 cm^3^ parietal WM voxel outlined in white on the patient in A; lower spectra was obtained from patient in B). Spectra were processed with LCModel (Stephen Provencher Inc., Oakville, Ontario, Canada, LCModel Version 6.1-4F). Note that the light grey line represents the acquired spectrum while the black line represents the model spectrum fit by LCModel. Metabolite concentrations were corrected for the varying fractions of cerebrospinal fluid in the selected ROIs.

**Figure 2:**
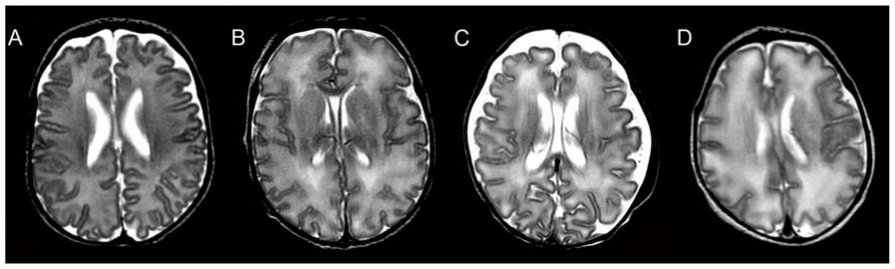
T2-weighted images for each neonate were rated for DEHSI. Examples of cases rated as “within normal limits (WNL)” (A), mild DEHSI (B), moderate DEHSI (C) and severe DEHSI (D) are depicted above. All 108 cases were rated independently for DEHSI by two investigators who used a modified version of the scale by Maalouf and colleagues (Table S1) [11].

Conventional MRI scans (T1-, T2- and Diffusion-weighted sequences) for all studies were independently reviewed by two investigators (JLW, AP) and scored for the presence of both pWMLs (defined as punctate T1-hyperintense lesions in the periventricular and intermediate white matter; see Figure 1A) and DEHSI (defined as high signal on T2-weighted MR images in the cerebral white matter and scored on a 4 point scale: 0/within normal limits, 1/mildly increased, 2/moderately increased and 3/severely increased (Figure 2; see also Table S1).

### Data acquisition

MRI studies were acquired under clinical indications (most often to assess brain morphology and injury following preterm birth) on a GE 1.5T (Signa LX, GE Healthcare, Milwaukee, WI) MR System using a customized neonatal transmit-receive head coil. Some studies were conducted using an MR compatible incubator; however, the majority of the studies were conducted with the neonate wrapped in a blanket and secured in the MR scanner with appropriate physiological monitoring equipment. Per clinical protocol, most infants were sedated with choral hydrate throughout the MR scan. Conventional imaging studies were acquired contemporaneously with the MRS studies and included a coronal SPGR sequence (TE = 6 ms; TR = 25 ms, FOV = 18 cm; matrix = 256×160) or axial and sagittal T1-weighted FLAIR sequences (TE = 7.4, TR = 2100; TI = 750; FOV = 20 cm; Matrix = 256×160), axial T2-weighted FSE sequence (TE = 85 ms, TR = 5000 ms, FOV = 20 cm, matrix = 320×160 or 256×128) and a diffusion-weighted sequence (TE = 80; TR = 10000; FOV = 22 cm; Matrix = 128×128; slice thickness = 4.5 mm, spacing 0 mm). ^1^H spectra were acquired from a single voxel (approximately 3 cm^3^) placed in the parietal white matter dorsolateral to the trigone of the lateral ventricle in the left hemisphere by use of a point resolved spectroscopy (PRESS) sequence with a short echo time (TE) of 35 milliseconds (ms), a repetition time (TR) of 1.5 seconds, 128 signal averages, and a total acquisition time for each spectrum of approximately five minutes, including scanner adjustments.

### Metabolites Analyzed

We focused our hypothesis-driven analyses on glutamate and glutamine. Nevertheless, to be consistent with prior studies of cerebral metabolism using MRS, we also analyzed four additional metabolites, including markers of neuronal/oligodendroglia and axonal integrity (N-acetyl aspartate, NAA), energy metabolism (creatine, lactate), and membrane integrity (choline).

### Data processing

Consistent with prior publications from our laboratory and elsewhere, absolute concentration for each of the six metabolites was quantitated from the MRS spectra using LCModel software (Stephen Provencher Inc., Oakville, Ontario, Canada, LCModel Version 6.1-4F) [26], [28], [29], [30], [31]. LCModel is an automated, user-independent software that quantitates the concentration of individual metabolites by fitting a library of concentration-calibrated model spectra to the acquired spectra. This method exploits the full spectroscopic information of each metabolite and not just isolated resonances. Therefore, it allows for a more accurate quantitation of metabolites with overlapping peaks (such as glutamate and glutamine; for more details, see below) than methods that fit lines only to the major peaks of spectra. Acquired spectra and LCModel fits were reviewed by JLW and SB for artifacts and other errors in model fitting. As noted above, MR spectra of low quality were removed by limiting the sample empirically to spectra with a linewidth (measure of field homogeneity) of <5 Hz and SNR≥5 [31]. The removal of spectra of poor quality limits the potential for inaccurate measurements of metabolite concentration at low SNR [28]. Of note, only metabolites that could be reliably measured using a Cramer Rao bounds of less than 50% (as calculated by LCModel) were used, including that of glutamate and glutamine.

Per protocol, metabolite concentrations were also corrected for the varying fractions of cerebrospinal fluid and tissue water content in the parietal white matter region of interest [32]. Furthermore, for absolute quantitation, the signal from unsuppressed water was used as the internal concentration reference. The water content of the developing human brain is known to change rapidly from approximately 92% in premature white matter to approximately 85% in the older infants within our population [33], [34], [35]. Our approach is to use the T2 relaxation time of tissue (which can be readily and accurately measured) to estimate water content from a look-up table. Several groups have independently shown that the T2 relaxation time correlates with water content [36], [37].

### Quantitation of glutamate separate from glutamine

The quantitation of glutamate and glutamine can be challenging as both molecules exhibit complex proton spectra with overlapping signals. However, glutamate and glutamine spectra are not identical, and by analyzing the full spectroscopic information of all metabolites simultaneously, LCModel allowed for reliable separation of glutamate and glutamine at 1.5T. This is underscored by the fact that, metabolically, we were able to demonstrate the expected compartmentalization of glutamate and glutamine in neurons and astrocytes, respectively (Table 1) [20], [21].

**Table 1:** Partial correlations among concentrations of each of the six metabolites, controlling for age (PCA).

	Glutamate	Glutamine	NAA	Creatine	Lactate	Choline
**Glutamate**	1.0					
**Glutamine**	**.255**	1.0				
**NAA**	**.470**	−.031	1.0			
**Creatine**	**.511**	.102	**.730**	1.0		
**Lactate**	.091	.160	**−.219**	−.148	1.0	
**Choline**	**.376**	**.252**	**.338**	**.665**	.032	1.0

Significant correlations are in **bold**. Note the correlations among glutamate, glutamine, and NAA, which provide convergent validity of our quantitation of glutamate separate from glutamine: glutamate and NAA are both synthesized in the mitochondria of neurons, whereas glutamine is synthesized in astrocytes. The pattern of cross-correlation is consistent with the compartmentalization of glutamate and glutamine in neurons and astrocytes, respectively.

### Statistical analyses

All statistical analyses were carried out in SPSS (Version 19, IBM Corporation). For the primary analyses, comparisons across the 108 preterm infants stratified independently with regard to both pWMLs and DEHSI, metabolite concentrations were compared across groups using MANCOVA, controlling for age at the time of the scan. Posthoc analyses (ANCOVA and pairwise comparisons as appropriate) were used to assess group differences for individual metabolites.

## Results

### White matter metabolism in neonates with and without pWMLs

**Figure 3:**
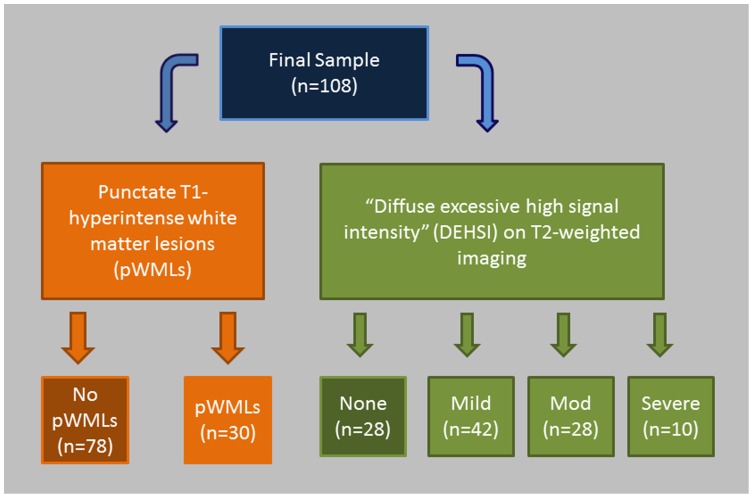
Cases were stratified independently for analyses of pWMLs and DEHSI. Depicted above is a schematic diagram demonstrating how the 108 preterm infants were stratified with regard to both pWMLs and DEHSI following independent evaluation by two investigators and subsequent consensus classification for cases discrepant between the raters with regard to pWMLs (n = 2) or DEHSI (n = 13). Overall kappas for pWMLs and DEHSI were 0.95 and 0.84, respectively.

One hundred and eight preterm neonates met the inclusion criteria (mean gestational age at birth: 31.0 weeks ±4.3; range 23–36 weeks), having been scanned during infancy (mean age at scan: 41.2 weeks ±6.0; range 25.7–60.7 weeks) (Figure 3). Across all 108 cases, inter-observer reliability (kappa) for MR-based ratings of pWMLs was 0.95. Following consensus review for the two cases discrepant for the pWML rating amongst the two observers, 30 cases had pWMLs (“pWML cases”, including 11 (37%) with evidence of additional cystic lesions in the white matter consistent with cavitated PVL). Seventy-eight cases did not have pWMLs (“non-pWML cases”) (see also Figure 1).

Overall, there were significant differences in the concentration of several metabolites among preterm infants with and without pWMLs (Multivariate F [6,100] = 3.455, *p* = 0.004). Univariate analyses indicated that glutamine was significantly increased by 29% (F [1,105] = 9.688, *p* = 0.002) and NAA was significantly decreased by 24% (F [1,105] = 9.652, *p* = 0.002) in the pWML cases compared to the non-pWML cases (Table 2). Parallel results were obtained when the ratio of each metabolite (glutamate, glutamine, NAA, lactate and choline) to creatine was used in the multivariate analysis in place of the absolute concentration (Table S2).

**Table 2:** Mean metabolite concentrations (mmol/kg) [standard error].

	pWML cases (n = 30)	Non-pWML cases (n = 78)	*p*-value^a^
Glutamate	**5.04 [0.38]**	**5.58 [0.24]**	**0.239**
Glutamine	**6.34 [0.35]**	**5.04 [0.22]**	**0.002**
NAA	**3.85 [0.20]**	**4.60 [0.13]**	**0.002**
Creatine	**4.96 [0.17]**	**5.30 [0.10]**	**0.082**
Lactate	**0.93 [0.14]**	**0.78 [0.08]**	**0.334**
Choline	**2.35 [0.08]**	**2.40 [0.05]**	**0.543**

for pWML cases and non-pWML cases. Mean values above are adjusted for age.

a *p*-values listed are derived from the univariate contrasts controlling for PCA.

### White matter metabolism in relation to DEHSI

Overall, there was generally good agreement between the two investigators with regard to the radiologic classification of DEHSI (kappa = 0.84). There was, however, mild disagreement on 10 of the cases scored as a 1 (mildly increased) or 2 (moderately increased). Because the analyses of DEHSI were performed in relation to severity ratings, the disputed cases (n = 13) were re-reviewed by the two investigators together and consensus ratings were used for all subsequent analyses.

Overall, there was no difference in the concentrations of all six metabolites relative to the DEHSI classification (F [18, 300] = 1.397, *p* = 0.131). However, because we had lower statistical power to detect differences in the multivariate analysis, and because we *a priori* hypothesized that the metabolites would be altered in the setting of DEHSI, we carried out univariate analyses (ANCOVA) to test whether the metabolites, individually, were altered in relation to DEHSI. Across all of the univariate analyses, only lactate differed among the four groups (F [3,103] = 5.569, *p* = 0.001). There were no differences in the concentration of glutamate or glutamine relative to the DEHSI classification (*p*'s>0.25) (Table 3).

**Table 3:** Mean of metabolite concentrations (mmol/kg) [standard error] across DEHSI subgroups.

	No DEHSI (n = 28)	Mild DEHSI (n = 42)	Moderate DEHSI (n = 28)	Severe DEHSI (n = 10)	*p*-value^a^
Glutamate	**5.95 [0.40]**	**5.31 [0.32]**	**5.22 [0.40]**	**5.02 [0.67]**	**0.499**
Glutamine	**5.99 [0.38]**	**5.05 [0.31]**	**5.38 [0.38]**	**5.28 [0.63]**	**0.314**
NAA	**4.56 [0.22]**	**4.59 [0.18]**	**4.18 [0.22]**	**3.69 [0.36]**	**0.094**
Creatine	**5.42 [0.17]**	**5.29 [0.14]**	**5.03 [0.17]**	**4.73 [0.29]**	**0.146**
Lactate	**1.01 [0.13]**	**0.61 [0.11]**	**0.69 [0.13]**	**1.51 [0.22]**	**0.001**
Choline	**2.48 [0.08]**	**2.37 [0.06]**	**2.31 [0.08]**	**2.38 [0.13]**	**0.513**

Mean values are adjusted for age. Multivariate F was not significant (F[18,300] = 1.397, *p* = 0.131). However, as noted in the main body of the text, given an *a priori* hypothesis that the concentrations of individual metabolites would be altered in relation to DEHSI, univariate analyses were conducted.

a *p*-values listed are derived from the univariate contrasts controlling for PCA.

**Figure 4:**
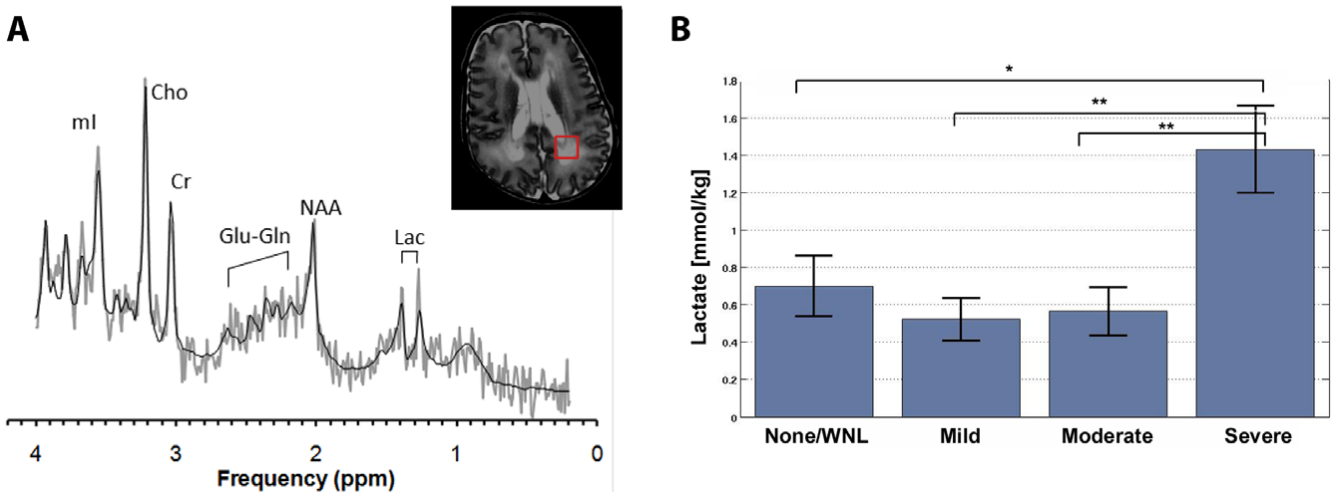
Lactate was elevated in preterm infants with severe DEHSI. On the left the MRS spectra obtained from the parietal white matter region of interest (red square, see inlet) demonstrates lactate. On the right, marginal means (controlling for PCA) are plotted for each DEHSI group for the subsample of infants ≥37 weeks PCA. Lactate was elevated in the severe cases, relative to the other three groups. * p<.05; ** p<.01. WNL = within normal limits.

Posthoc analyses revealed that, in addition to the infants with severe DEHSI, the lactate concentration was significantly elevated in preterm infants with no DEHSI compared to infants with mild or moderate DEHSI. To determine whether this stemmed from the inclusion of preterm infants who were not yet term-equivalent in age, for whom slight lactate elevations have been reported in otherwise healthy preterm infants [25], [26], [27], we repeated the DEHSI analysis with regard to lactate, restricting the sample to infants who were at least term-equivalent age (≥37 weeks PCA; n = 87) at the time of the MRI. Results from this ANCOVA again demonstrated significant differences amongst the DEHSI groups (F [3, 82] = 4.506, *p* = .006). Posthoc analyses revealed an elevation in lactate for the infants with severe DEHSI, relative to infants without DEHSI [*p* = .015] or with mild or moderate DEHSI [*p*'s = .001]. Moreover, lactate concentrations among the infants classified as without evidence of DEHSI, mild DEHSI and moderate DEHSI were not significantly different (Figure 4).

## Discussion

In this study, using clinically-available ^1^H-MRS, we observed different patterns of abnormal cerebral metabolism in the parietal white matter in association with the two most predominant MRI-defined patterns of evolving WMI in preterm infants, i.e., pWMLs and DEHSI. Our results indicate, for the first time, that glutamine concentrations are abnormally elevated in association with pWMLs, but not in association with DEHSI, likely reflecting the disruption of glutamate-glutamine homeostasis in focally necrotic white matter in subacute stages of evolution, but not in diffuse white matter gliosis without necrotic foci. These results suggest that MRI/MRS may not only be a useful tool for identifying WMI in the preterm neonate, but also for differentiating the underlying metabolic pathogenesis of different patterns of WMI.

In animal models of WMI, a rise in extracellular glutamate has been observed in the acute period (i.e., first two hours) following hypoxia-ischemia [38], and glutamate receptor antagonists administered pre- or immediately post-insult attenuate histologically-defined patterns of WMI, as well as resultant motor deficits [17] [18]. In this study, the peak (acute) window of elevated glutamate was potentially missed, in large part because the periods in which the initial insult is most likely sustained (i.e. shortly after birth or during periods of clinical instability) are not the time points when most diagnostic MR imaging is carried out in critically ill premature infants. Thus, most likely, we are measuring metabolite concentrations during the subacute phase of evolving injury characterized by pWMLs with macrophagocytic infiltration. Consequently, the finding of altered glutamatergic metabolism in pWMLs further suggests that glutamate toxicity may persist past the acute onset of injury, such that interventions with protective agents against this toxicity may be valuable beyond the acute phase when pWMLs and elevated glutamine concentrations are detected by MRI and MRS, respectively.

Our major finding of elevated glutamine concentration, but not glutamate, in association with pWMLs raises the question of the relationship of glutamine to glutamate toxicity. Although glutamine itself is not toxic to brain cells, and may even be neuroprotective [39], under conditions of hypoxia in cell culture or ischemia in rats, injured neurons are known to release mitochondrial glutaminase, the enzyme that converts glutamine to glutamate, and thereby lead to secondary elevations in extracellular glutamate levels [40], [41]. Moreover, elevated glutamine concentrations may reflect the temporal state of the WMI and the conversion of residual glutamate to glutamine, resulting in a change in the balance of the two amino acids relative to each other [42], [43], [44]. In addition, elevated glutamine concentrations may reflect ongoing inflammation, i.e., reactive gliosis, diffuse microglial activation and macrophage infiltration into necrotic foci, that is set in motion, at least in part, by excitotoxicity to pre-myelinating oligodendrocytes and/or axons [5]. In addition to normal astrocytes, glutamate is taken up by reactive astrocytes and macrophages in the inflammatory response to hypoxia-ischemia and/or infection via glutamate transporters for its conversion to glutamine [20], [21], [42], [43], [44]. It is uncertain if this protective detoxification process persists beyond the inciting elevation of glutamate itself; yet, it is known from neuropathologic studies that inflammatory cells (reactive astrocytes and microglia and macrophages) persist for days, weeks, and even months after the acute and inciting injury [45], [46]. Moreover, the EAAT2-glutamate transporter is upregulated in macrophages and reactive astrocytes at autopsy in infants with subacute and chronic PVL, supporting the interpretation of a role for the protective uptake of extracellular glutamate and its conversion to glutamine in the pathological process in preterm infants [47]. At the same time, the formation of glutamine by astrocytes requires ATP, and thus, it also requires that astrocytes be functioning metabolically at a sufficient level to generate ATP. Therefore, the data suggest that some aspects of astrocyte metabolism (e.g. glutamate uptake and glutamine formation) may be relatively normal in these infants.

In addition to the glutamate-glutamine cycle between neurons and astrocytes, a major consideration in the interpretation of the findings in this study is the role of glutamate or glutamine in other metabolic processes, which, if deranged, could likewise lead to elevated glutamine concentrations. Most notably, glutamine synthesis is the primary mechanism by which ammonia is detoxified in the brain. Consequently, elevated glutamine is observed in the brain in the setting of hepatic liver failure [48]. We excluded infants with clinical or laboratory evidence of liver failure (whether associated with congenital defect or acute clinical deterioration), hyperbilirubinemia, and inborn errors of ammonia metabolism. Thus, it is unlikely that the elevated glutamine concentrations in the preterm cases with pWMLs resulted from hyperammonia of peripheral origin. Yet, ammonia is also the byproduct of protein and amino acid catabolism in the brain, and it is possible that the elevated glutamine might be the net product of significant levels of cellular degeneration/death. We also observed a decrease in NAA in the parietal WM of infants with pWMLs. A product of mitochondrial metabolism in neurons, and possibly, pre-myelinating oligodendrocytes, NAA is often used as a biomarker to estimate the extent of cell damage or death [49], [50], [51], [52], [53], [54]. Accordingly, the elevation in glutamine coupled with a decrease in NAA in our cases with pWMLs may reflect damage or loss of axons and pre-myelinating oligodendrocytes in the subacute phase of injury, as shown in neuropathology studies of PVL [5], [55], [56].

The lack of an association of elevated glutamine concentrations and DEHSI in our preterm infants suggests that altered glutamatergic metabolism is not likely to be a major factor in its pathogenesis, despite the increase in the number and density of reactive astrocytes that putatively define this lesion. Elevated lactate levels were observed in the cases with severe DEHSI relative to cases with mild, moderate, or no DEHSI. Although the radiologic classification of DEHSI is most often applied along an ordinal scale, there may be qualitative differences between infants with severe DEHSI and infants with only mild or moderate DEHSI, a possibility consistent with data from recent longitudinal studies of infants with DEHSI assessed neurodevelopmentally between 2 and 3 years of age [57], [58]. In these studies, mild and moderate DEHSI show no relation to adverse neurodevelopmental outcome; however, severe DESHI is associated with an increased risk for neurodevelopmental delay [57], [58]. The finding of elevated lactate in association with severe DEHSI suggests that this pattern of WMI may be associated with energy failure leading to tissue acidosis that does not necessarily involve excitotoxicity.

## Conclusion

The results of our study suggest that MRS may provide a useful biomarker for excitotoxic WMI in preterm infants beyond the period of acutely and presumably transiently elevated levels of glutamate. Irrespective of the precise mechanism of elevated glutamine in pWMLs, its finding indicates a role for altered glutamatergic metabolism, reflected in an imbalance between glutamate and glutamine levels, in their pathogenesis. Thus, glutamine levels in WMI may prove clinically valuable in monitoring treatment response and early outcome in neonates treated with neuroprotective agents targeting excitotoxicity.

## Supporting Information

Table S1 **Guidelines for DEHSI ratings.** Wnl = Within normal limits. See Figure 2 for examples.
(DOCX)

Table S2 **Mean metabolite concentration for pWML cases and non-pWML cases expressed as a ratio relative to creatine.** Consistent with the results from the MANCOVA contrasting absolute concentration for the six metabolites, the overall multivariate F contrasting between the pWML cases and the non-pWML, controlling for PCA, was significant (F[5,101] = 4.502, *p* = 0.001). Univariate contrasts indicated a significant increase in the ratio of glutamine to creatine (F[1,105] = 15.905, *p*<0.001) and a significant decrease in the ratio of NAA to creatine (F[1,105] = 8.786, *p* = 0.004). Values above represent adjusted means (controlling for PCA) and [standard error].
(DOCX)
