# Supplementary material for: Altered Glutamatergic Metabolism Associated with Punctate White Matter Lesions in Preterm Infants
Source: PLoS One. 2013 Feb 26;8(2):e56880. doi: 10.1371/journal.pone.0056880 (PMC3582631; doi:10.1371/journal.pone.0056880)
Supplement: Table S1 — Guidelines for DEHSI ratings. Wnl = Within normal limits. See Figure 2 for examples. (DOCX) [file pone.0056880.s001.docx]

| DEHSI Rating | Descriptive classification |
| --- | --- |
| None/wnl | Uniform signal throughout the white matter, without evidence of high T2 signal in the periventricular or deep white matter |
| Mildly increased | Relatively circumscribed, but definitive areas of mildly increased T2 signal, often intermixed with areas of darker T2 signal within the periventricular white matter (i.e., “bands” of migrating cells). However, merely a “band” of high signal between areas of darker T2 signal, consistent with areas of immature white matter between migrating cells, was not sufficient to generate the classification of mild DEHSI. To receive a rating of mild DEHSI, the areas of excessive T2 signal either had to extend beyond the regions between the bands or there had to be some other evidence in the pattern of excessive T2 signal, such as asymmetry, indicating something beyond a pattern associated with “normal” development of the white matter. |
| Moderately increased | Larger regions of increased T2 signal generally “brighter” and of a greater spatial extent than that of above. Areas of darker T2 signal within the periventricular white matter (“bands”) were generally still visible, but were not required for classification. The spatial extent of excessive T2 signal may have been limited to the periventricular/deep white matter or it may have extended into the corona radiata or intragyral white matter. The key aspect was that the T2 signal was definitively brighter and greater in extent for cases rated moderate as compared to cases rated mild. |
| Severely increased | Large regions of increased T2 signal, markedly brighter than observed above and distributed through a large extent of the cerebral white matter, often extending into the intragyral white matter. Areas of darker T2 signal within the periventricular white matter (“bands”) were often not present, but their absence was not required for this classification. The key aspect was that the T2 signal was markedly bright. |
