# Supplementary material for: Altered Glutamatergic Metabolism Associated with Punctate White Matter Lesions in Preterm Infants
Source: PLoS One. 2013 Feb 26;8(2):e56880. doi: 10.1371/journal.pone.0056880 (PMC3582631; doi:10.1371/journal.pone.0056880)
Supplement: Table S2 — Mean metabolite concentration for pWML cases and non-pWML cases expressed as a ratio relative to creatine. Consistent with the results from the MANCOVA contrasting absolute concentration for the six metabolites, the overall multivariate F contrasting between the pWML cases and the non-pWML, controlling for PCA, was significant (F[5,101] = 4.502, p = 0.001). Univariate contrasts indicated a significant increase in the ratio of glutamine to creatine (F[1,105] = 15.905, p<0.001) and a significant decrease in the ratio of NAA to creatine (F[1,105] = 8.786, p = 0.004). Values above represent adjusted means (controlling for PCA) and [standard error]. (DOCX) [file pone.0056880.s002.docx]

| Ratio | pWML cases (n=30) | Non-pWML cases (n=78) | *p*-value |
| --- | --- | --- | --- |
| Glutamate/creatine | 0.99 [0.06] | 1.01 [0.04] | 0.862 |
| Glutamine/creatine | 1.16 [0.07] | 0.86 [0.04] | <0.001 |
| NAA/creatine | 0.77 [0.03] | 0.86 [0.02] | 0.004 |
| Lactate/creatine | 0.28 [0.04] | 0.21 [0.03] | 0.133 |
| Choline/creatine | 0.52 [0.01] | 0.50 [0.01] | 0.220 |
